# Development of a Genomic Instability-Derived lncRNAs-Based Risk Signature as a Predictor of Prognosis for Endometrial Cancer

**DOI:** 10.7150/jca.65581

**Published:** 2022-04-11

**Authors:** Xiaojun Wang, Lei Ye, Bilan Li

**Affiliations:** Department of Gynaecology, Shanghai First Maternity and Infant Hospital, School of Medicine, Tongji University, Shanghai, 200092, China.

**Keywords:** Endometrial cancer, genome instability, long non-coding RNA, prognosis

## Abstract

Endometrial cancer (EC) ranks fourth in the incidence rate among the most frequent gynaecological malignancies reported in the developed countries. Approximately 280,000 endometrial cancer cases are reported worldwide every year. Genomic instability and mutation are some of the favourable characteristics of human malignancies such as endometrial cancer. Studies have established that the majority of genomic mutations in human malignancies are found in the chromosomal regions that do not code for proteins. In addition, the majority of transcriptional products of these mutations are long non-coding RNAs (lncRNAs). In this study, 78 lncRNA genes were found on the basis of their mutation counts. Then, these lncRNAs were investigated to determine their relationship with genomic instability through hierarchical cluster analysis, mutation analysis, and differential analysis of driving genes responsible for genomic instability. The prognostic value of these lncRNAs was also assessed in patients with EC, and a risk factor score formula composed of 15 lncRNAs was constructed. We then identified this formula as genome instability-derived lncRNA-based gene signature (GILncSig), which stratified patients into high- and low-risk groups with significantly different outcome. And GILncSig was further validated in multiple independent patient cohorts as a prognostic factor of other clinicopathological features, such as stage, grade, overall survival rate. We observed that a high-risk score is often associated with an unfavourable prognosis in patients with EC.

## 1. Introduction

Endometrial cancer (EC), which is the most frequently reported gynaecological malignancy, ranks fourth in terms of the incidence rate in developed countries. Approximately 280,000 cases are reported worldwide every year 1. EC majorly affects postmenopausal women, and the incidence rate spikes are observed in women aged between 55 to 65 years old 1. Clinically, 80% of patients with EC present with abnormal vaginal bleeding, which benefits the early diagnosis and treatment and has led to an improvement of the 5-years survival rate of EC patients 2. However, there are 20% of cases presented with metastasis of pelvic cavity and lymph node, and about 10% of cases presented with distant metastasis at diagnosis 3. The prognosis varies according to the stage of EC. The 5-years survival rate of EC patients at stage I was 80%-90%, but it declined to about 20% in EC patients at stage IV 4. Hence, novel strategies are warranted to assess the prognosis of patients with EC and evaluate the clinical outcomes.

Genomic instability and mutation are common characteristics in human malignancies. 5. Genomic changes occur through several pathway such as single or minority nucleotide mutations and acquisition or loss of a whole chromosome, probably leading to abnormal division, multi-nucleation, and trimeric mitosis 6, 7. Different types of human malignancy exhibit different somatic mutation spectrums, corresponding to different numbers of gene mutations, indicating the tissue-specific or cell-specific tumourigenic mechanisms 8, 9. In addition, as an evolutionary marker of human malignancy, genomic instability occurs mainly due to the mutation of DNA repair genes, which in turn promotes the progression of human malignancy and has been regarded as a key prognostic factor^10^-^12^. Hence, intensive study of the molecular features of genomic instability in various types of malignancies and investigating their clinical significance are essential.

Several genomic mutations in human malignancies are found in the chromosomal regions that do not code for proteins. In addition, a majority of transcriptional products of these mutations are long non-coding RNAs (lncRNAs) 13. Evidence accumulated during the past few decades suggests the involvement of lncRNAs in gene regulation, proliferative capability, migratory behaviour, and genome stability. These multi-functional regulatory activities make lncRNAs a valuable signature factor for human malignancies 14. Notably, lncRNAs associated with gene changes can promote tumour growth and affect genomic stability. For instance, a novel lncRNA CCAT2 containing the rs6983267 SNP, whose expression level is abnormally high in microsatellite stable (MSS) colorectal cancer, has been shown to promote cancer progression, metastatic behaviour, and chromosomal instability 15. Another study that performed somatic copy number changes (SCNAs) of lncRNAs showed that the lncRNAs of genomic changes or localized changes targeting genes for tumourigenic lncRNAs^16^. In addition, cancer related lncRNAs have been shown to contribute to increased genome instability and malignant behavior 17. Conversely, some lncRNAs including NORAD, CUPID1, CUPID2, and DDSR1 facilitate the repair of DNA damage and exhibit genome stability 18-20. Although lncRNAs play a key role in the regulation of genome stability, the clinical significance and underlying mechanism of lncRNAs related to genomic instability (GILncRNAs) in EC were not completely understood.

In this study, we retrieved the lncRNA data and mutation data of patients with EC from the human malignancy genome atlas (TCGA) database. In addition, we assessed the prognostic value of the established GILncSig associated with genomic instability in EC. It is hypothesized that GILncSig has the potential to be utilized as a prognosis predictor in patients with EC. Overall, this study intended to assess the value of GILncSig as an independent prognostic predictor and provide an alternative assessment of genomic instability and human malignancy-related mortality risk.

## 2. Materials and Methods

### 2.1 Data retrieval and handling

The transcriptional profiles, clinical data, and somatic mutation profiles of patients with EC were obtained from the TCGA database (https://portal.gdc.human malignancy.gov/). The expression levels of lncRNAs and mRNAs in EC samples were extracted from the transcriptional data. The lncRNAs from the expression profile were extracted, the expression values of lncRNAs with the same Symbol were averaged, and the genes whose expression level was less than 30% were removed. Then, we integrated of the expression data and mutation data to obtain the intersection sample information. Finally, the expression matrix of 499 samples and 3527 lncRNAs was obtained for subsequent analysis.

### 2.2 Screening of lncRNAs Related to Genome Instability

To identify genome instability-associated lncRNAs, a hypothesis mutator-derived computational frame combining lncRNA expression profiles and somatic mutation profiles in a tumour genome: (i) the cumulative number of somatic mutations for each patient was computed; (ii) patients were ranked in decreasing order of the cumulative number of somatic mutations; (iii) the top 25% of patients were defined as genomic unstable (GU)-like group, and the last 25% were defined genomic stable (GS)-like group; (iv) expression profiles of lncRNAs between the GU group and GS group were compared using significance analysis of the ʻLimmaʼ package of R software to analyse GILncRNAs with different expression levels, where the threshold was |logfc| > = | log 1.3 | and the P value was <0.05.

### 2.3 Construction of the lncRNA-mRNA network and functional enrichment of mRNA

Based on the interactions data from the RNAInter database (http://www.rna-society.org/raid/download.html), Cytoscape was used for visualisation to extract the mRNAs interacting with GILncRNAs. Furthermore, the functional enrichment of interacting mRNAs was analysed, and the cluster profiler was utilised for the pathway enrichment analysis. We utilised org. HS. Eg. DB to transform gene names and GOplot & ggplot 2 to visualise the pathways.

### 2.4 Hierarchical Clustering based on GILncRNAs

According to the GILncRNAs-lncRNAs in all the samples, the Consensus Cluster Plus package of R software was utilized to cluster the samples for unsupervised analysis. The clustering method used was K-means, and the distance function utilized was Euclidean. The variation of the two sample sub-types was counted. The group with a high variation was called the GU-like group, whereas the lower group was called the GS-like group, and the two sub-types based on the stability of the genome were finally determined. Survival of the two sub-types was analysed using the 'Survival' and 'Survminar' packages of R software, and the KM curve was drawn. The heat map of GILncRNAs expression in two sub-types was drawn using R-package complex heatmap.

### 2.5 Establishment of GILncRNAs-Based Prognostic Analysis Methods

The samples were allocated into a training set and a testing set (the ratio of samples in the training set and testing set was 7:3). The Chi-square test was used to ensure that no deviation is present in the division of the training data set and test data set. Then, 'Survival' and 'Survminar' packages of R software were utilized to conduct the univariate Cox regression analysis. LncRNAs with Cox P < 0.05 were considered as the candidate genes with prognostic values. Then, the least absolute shrinkage and selection operator (LASSO) regression algorithm was utilized to screen candidate GILncRNAs. The LASSO Cox regression was then used to select variables for constructing the signature and provide coefficients. The risk score was calculated using the following formula: risk score = expression level of lncRNA1 × β1 + expression level of lncRNA2 × β2+ … + expression level of lncRNAn × βn, where risk score is a measure of prognosis of patients with EC, and β is the regression coefficient for each variable. The risk score of each patient was calculated according to the risk characteristics, and then, they were divided into two groups (high-risk and low-risk) based on the risk score. We utilized the Kaplan-Meier method to plot the curve of survival of patients in the two groups. Furthermore, the log-rank test was utilised to assess the survival of patients, P < 0.05. Finally, the GILncSig risk model was employed in the testing set and TCGA set to assess its function.

### 2.6 Prognosis Prediction and Clinical Stratification Analysis

To examine the potential role of GILncSig as an independent predictor of other crucial clinicopathological parameters, the univariate and multivariate Cox regression analyses were conducted using the 'Survival' package of R software. A P value of <0.05 was considered to signify statistical significance. Then, the clinical stratification analysis was performed to evaluate the value of GILncSig for predicting prognosis in patients with EC. According to the clinical parameters including age, the patients in The Cancer Genome Atlas (TCGA) were divided into subgroups according to the age (≥ 60 years), and disease course (stage I-II and stage III-IV). Based on the median value of the GILncSig score, cases in each clinical subgroup were further allocated into two groups (high-risk and low-risk). We then performed the Kaplan-Meier analysis and log-rank test to analyse the survival rates.

### 2.7 Establishment and Verification of a Nomogram Scoring System

Nomograms were used to display the results of Cox regression directly. According to the regression coefficients of all the independent variables, the scoring standard was set, and the total score of each patient was calculated, then the probability of each patient's prognosis time was calculated using the conversion function between the score and the prognosis probability. The nomograms were mainly drawn using the 'RMS' and 'sarviva' packages of R software. Firstly, the Cox proportional hazard regression model was constructed with CPH, and then, the Survival function was utilized to calculate the survival probability. Finally, the nomogram function tree was utilized to construct the nomograms, which showed as the plot, and the correction curve and time-dependent ROC prediction curve were assessed.

### 2.8 Statistical Analysis

Chi-square test and Mann-Whitney U test were utilized to assess differences in the classification and quantitative data. A 2-tailed P value of < 0.05 denoted statistical significance. R version 4.0.2 (Institute of statistics and mathematics, Vienna, Australia 4) was compared by visual and statistical Analysis.

## 3. Results

### 3.1 Identification of Genome Instability-Related lncRNAs

**Figure 1:**
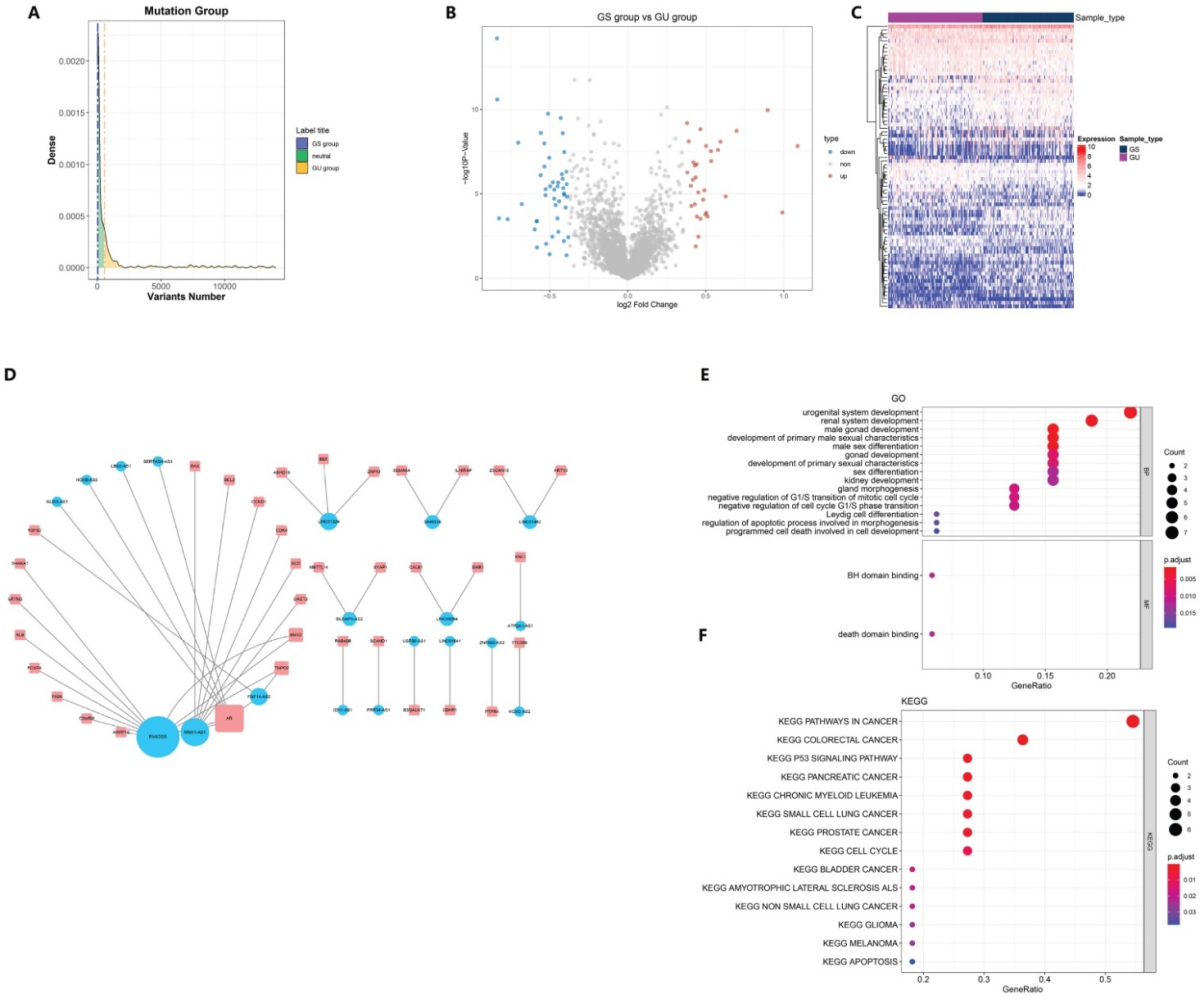
Screening and functional annotation of genomic instability-related lncRNAs. (A) screening of differentially expressed lncRNAs as genomic instability-related lncRNAs (GILncRNAs), (B) volcanic areas of 78 tunas, (C) unsupervised hierarchical clustering analysis of 499 EC patients. The higher one was designated as the genomic instability-like cluster (GU-like), and the lower one was designated as the genomic stability-like cluster (GS-like) (D). The expression network of GILncRNAs and their related mRNAs were analyzed. The orange and blue circles represent GILncRNAs and protein encoded mRNAs, respectively. It is necessary to draw the names of GILncRNAs and their highest co-expression mRNAs (E) mRNAs and GO enrichment analysis (P < 0.05), (F) of lncRNAs co-expressed through Pearson's correlation coefficient in the network. Functional enrichment analysis of lncRNAs co-expressed through mRNAs by KEGG (P < 0.05).

Of the 499 samples, 130 EC patients with the highest mutation rate were assigned to the GU-like group, whereas 125 patients with the lowest mutation rate were assigned to the GS-like group (Fig. 1A). Then, the differentially expressed genes (DEGs) of the two groups were detected and 78 lncRNAs were found, with 32 lncRNAs up-regulated and 46 lncRNAs down-regulated (Fig. 1B). To determine whether the differentially expressed lncRNAs reflected the genomic instability of the patients, we performed an unsupervised hierarchical clustering assay on the 78 lncRNAs. All 499 cases were divided into two groups with a significant difference in their mutation count (Fig. 1C). Next, we explored the potential function of GILncRNAs through the co-expression analysis and GO enrichment analysis. The lncRNA-mRNA co-expression networkwas used to show the relationship between lncRNAs and mRNAs (Fig. 1D). A total of 43 pairs of interacting GILncRNAs and mRNAs were identified, indicating that GILncRNAs are tightly correlated with the regulation of mRNAs expression. GO analysis of GILncRNAs-associated genes revealed that DE-lncRNA with mRNAs in this network are significantly associated with Binding to a Bcl-2 homology (BH) and death domain binding in molecular function (MF) as well as mitotic cell cycle regulation in biological process (Fig. 1E). All the aforementioned factors are believed to be associated with genome stability. Based on the KEGG pathway analysis of lncRNA-related protein coding genes (PCG), 39 most enriched pathways were identified and the most of them were found to be related to the genome stability factors such as cell cycle regulators and malignancies (Fig. 1F). Collectively, these results suggested that 78 differentially expressed lncRNAs are associated with genome stability. In addition, the expression levels of these lncRNAs might compromise the cellular genome stability by disrupting the equilibrium of lncRNA-associated PCG modulatory web, thus tampering with the regular repairing pathways for genomic damage and causing an increased genome instability.

### 3.2 Hierarchical Clustering based on GILncRNAs

**Figure 2:**
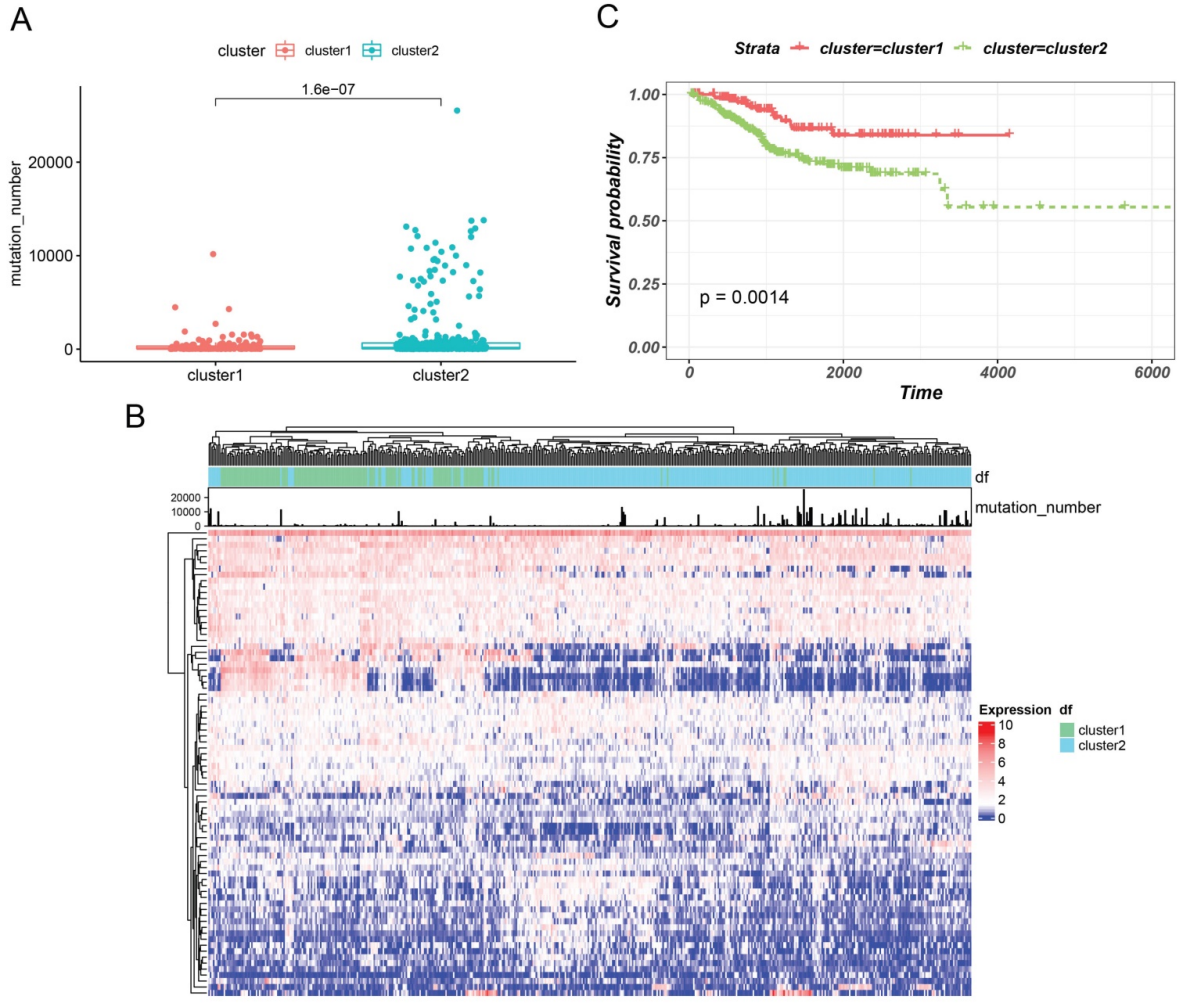
Hierarchical clustering (a) based on GILncRNAs was used to cluster all the samples in an unsupervised manner. The higher one was designated as the genomic instability-like cluster and the lower one as the genomic stability-like cluster. (B) The unsupervised hierarchical cluster analysis heat map of 499 EC patients revealed the Kaplan-Meier curve of the mutation number, (C) the class GU group, and class GS group.

Based on GILncRNAs, 499 EC patients were divided into two groups through unsupervised clustering (154 patients in Cluster 1 and 345 patients in Cluster 2). We defined the group with a high mutation number as GU-like and the other group as GS-like. As shown in Figures 2A and 2B, the number of mutations in cluster 2 appeared to be significantly higher than that in cluster 1 (P = 1.6e-07). Hence, cluster 2 was defined as the GU-like group, and cluster 1 was defined as the GS-like group. Then, survival of the two subtypes was analysed. The survival curve revealed remarkable differences , with the GU-like group showing poor prognosis compared to the the GS-like group (P=0.0014). These results indicated that genome instability is strongly correlated with patient's survival.

### 3.3 Screening of the GILncSig and Predictability Evaluation

**Figure 3:**
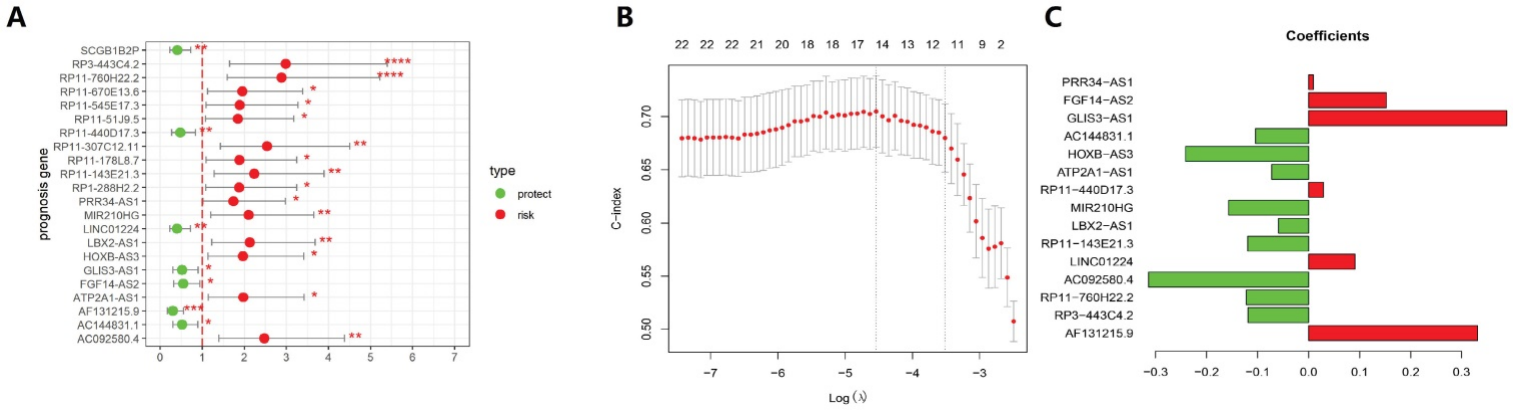
Establishment of prognosis signature in EC utilising GILncRNAs in the training set. (A) A total of 22 GILncRNAs correlating with the overall survival of patients with EC were plotted. (B) The distributing pattern of the LASSO coefficient. (C) The distributing pattern of the LASSO coefficient of 15 most significant GILncRNAs.

The 499 EC cases were randomly allocated into a training group and a test group with the ratio as 7:3. A total of 22 lncRNAs that were tightly associated with the survival rates in the training set were examined. Of these 22 lncRNAs, 7 lncRNAs were protective factors, whereas 15 lncRNAs were risk factors (Fig. 3A). Furthermore, 22 prognosis-related lncRNAs identified through Cox uni-variate regressions were selected for the LASSO regression. To construct the best model, the minimum lambda value, which is lambda.min, was selected through cross-validation, and then, 15 more significant lncRNAs from the 22 lncRNAs were selected to construct a human malignancy-related prognostic risk score model (P < 0.05, Figure 3B and 3C). According to the optimised model, the following formula was utilised to calculate the risk score: Risk score = 0.331 × AF131215.9 - 0.119 × RP3 - 443C4.2 - 0.123 × RP11 - 760H22.2 - 0.314 × AC092580.4 + 0.091 × LINC01224 - 0.119 × RP11 - 143E21.3 - 0.059BX2 - AS1 - 0.157 × MIR210HG + 0.029 × RP11 - 440D17.3 - 0.073 × ATP2A1 - AS1 - 0.241 × HOXB - AS3 - 0.104 × AC144831.1 + 0.389 × GLIS3 - AS1 + 0.152 × FGF14 - AS2 + 0.009 × PRR34 - AS1. The risk score was used to categorise the cases into two groups (high-risk and low-risk groups) for the subsequent analysis. In the equation of GILncSig, six lncRNAs (PRR34-AS1, FGF14-AS2, GLIS3-AS1, RP11-440D17.3, LINC01224, and AF131215.9) with positive coefficients were regarded as risk factors, and the abnormal up-regulation of these genes correlated with poor prognosis. On the other hand, another eight lncRNAs (AC144831.1, HOXVB-AS3, ATP2A1-AS1, MIR210HG, LBX2-AS1, AC092580.4, RP11-760H22.2, and RP3-443C4.2) with negative coefficients were considered as protective factors, whose up-regulation correlated with better outcomes.

**Figure 4:**
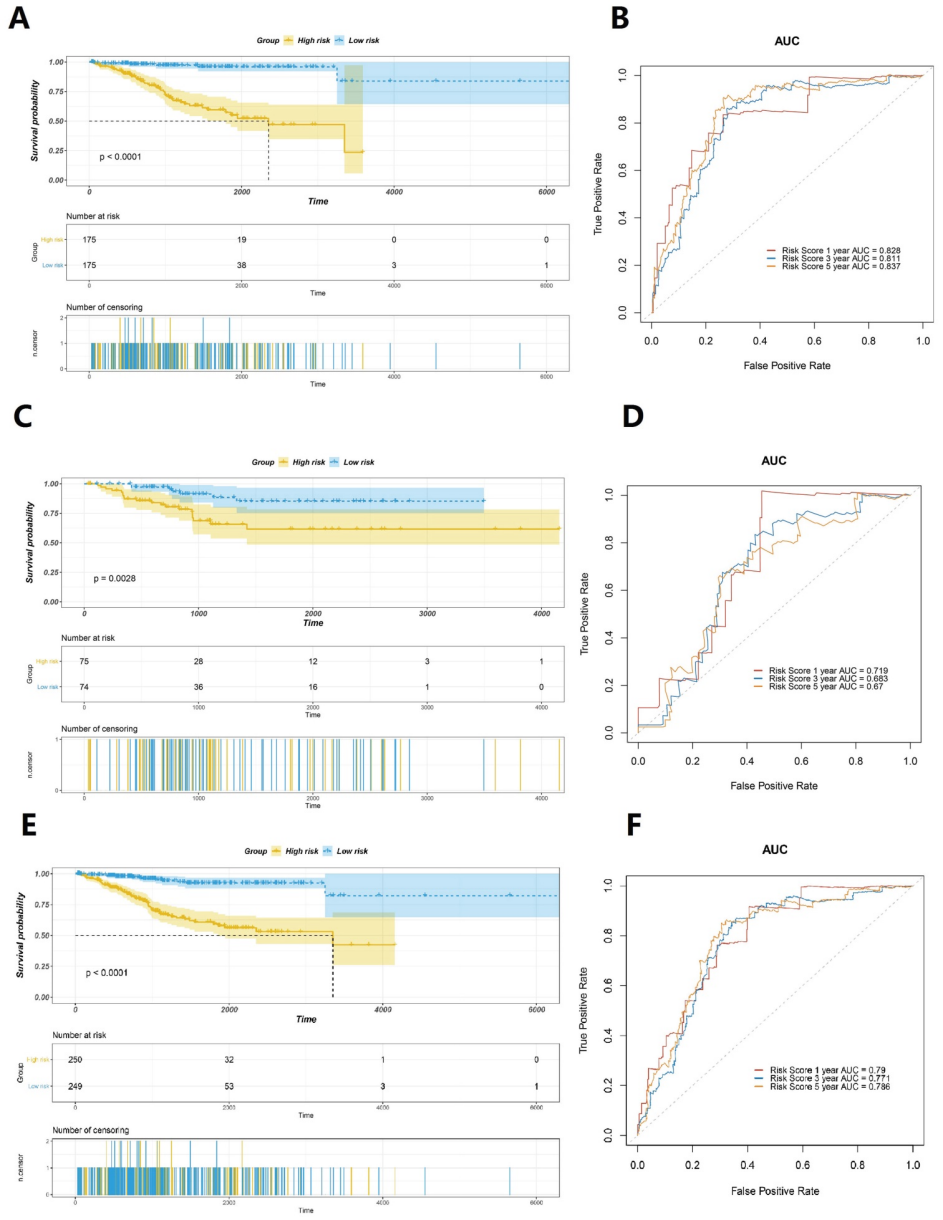
Evaluation of the prognostic significance of GILncSig in EC. Survival curves of the patients with EC were plotted by utilising the Kaplan-Meier method in the training set (A), the testing set (C), and the TCGA set (E). Cases in the low-risk group showed a more favourable prognosis. ROC curves to predict 1-year survival in the training set (B), the testing set (D), and the TCGA set (F).

According to the calculated risk score, cases with scores greater than the median were categorised as the high-risk group, whereas cases with scores ≤ the median were categorised as the low-risk group. The results revealed that cases in the low-risk group had a better prognosis than those in the high-risk group (Fig. 4A). The area under curve (AUC) values of the ROC curves in the training set for the 1-year, 3-year, and 5-year survival prediction of risk scores were 0.828, 0.811, and 0.837, respectively (Fig. 4B). To verify the accuracy of predicting the survival rate using risk scores, we calculated the risk scores of the test set and the whole TCGA set and plotted the ROC curves. In the test set, the survival time of the low-risk group was observed to be longer than that of the high-risk group (Fig. 4C). The AUC values of the ROC curves in the training set for the 1-year, 3-year, and 5-year survival prediction of risk scores were observed to be 0.719, 0.683, and 0.67, respectively (Fig. 4D). The results obtained were similar to those in the entire TCGA dataset, which confirmed that patients with EC in the low-risk group exhibit significantly longer survival (Figure 4E). The time-dependent ROC curves analysis of the GILncSig yielded an AUC in the training set for the 1-year, 3-year, and 5-year survival prediction of risk score were 0.79, 0.771, and 0.786, respectively (Fig. 4F). All these findings suggested that the risk score is strongly associated with a great survival predictive significance.

### 3.4 Risk scores are associated with clinical features

**Figure 5:**
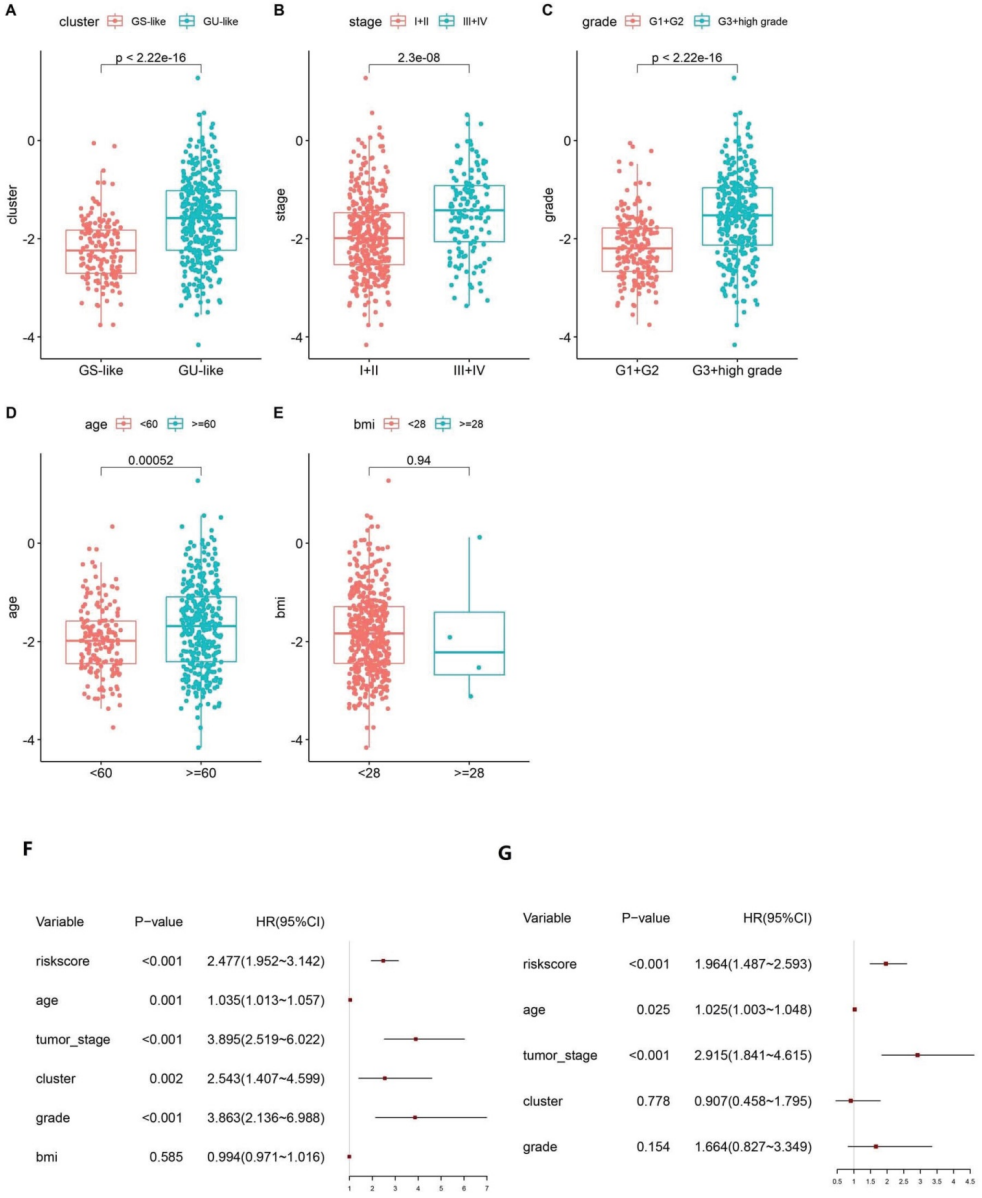
Boxplot of correlation between the risk score and GU-like or GS-like group, (A) tumour stage of the patients, (B) tumour grade of the patients, (C) age of the patients, (D) and BMI of the patients (E). Univariate (F) and multivariate (G) Cox regression analyses of the GILncSig and clinicopathological characteristics.

Based on the calculated risk scores, a correlation analysis with clinical features was performed. The risk scores in the GS-like/GU-like subgroups were found to differ significantly, with the risk scores being higher in the group with genomic instability (Fig. 5A). The risk scores were distributed differently in the various stages of EC and were higher in the stage III - IV group (Fig. 5B). Additionally, the risk scores were distributed differently in patients with a different grade and were higher in the G3 + high group than that in the G1 + G2 group (Fig. 5C). In addition, the risk score was distributed differently in the varied age groups, and the patients aged less than 60 years tended to have higher risk scores. However, no significant difference was observed between the BMI groups (Fig. 5D-E). Altogether, these findings verified the efficacy of GILncSig in predicting prognosis of patients with EC.

### 3.5 Assessment of the Independent Prognostic Value of GILncSig

**Figure 6:**
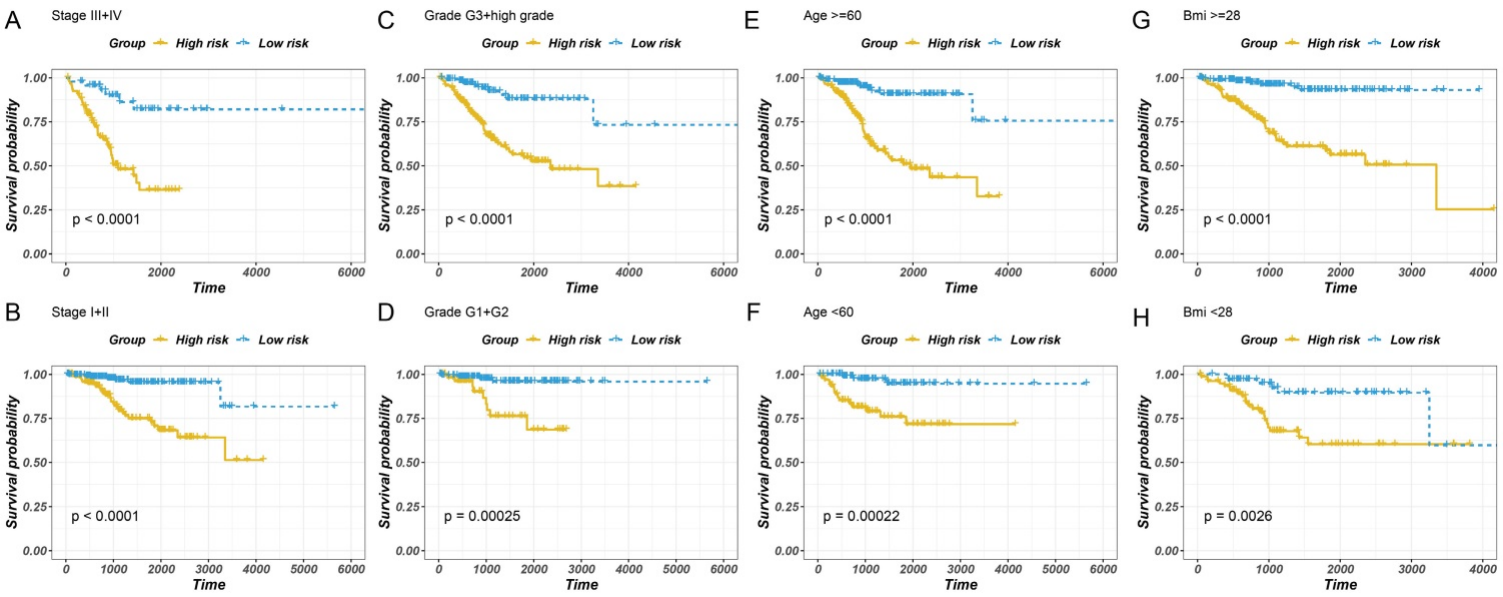
Stratified analysis of survival of patients with EC. The survival curves of patients with EC were plotted using the Kaplan-Meier method within six subgroups, including patients with the tumour stage III-IV (A), the tumour stage of I-II (B), the tumour grade of G3 + high (C), the tumour grade of G1 + G2 (D), age > 60 years (E), ≤6 0 years (F), BMI ≥ 28 (G) and BMI < 28 (H).

To examine the independent prognostic value of GILncSig, uni-variate and multivariate Cox regression analyses were performed on all the patients, and factors such as age, disease course, and GILncSig were included. The uni-variate analysis revealed that GILncSig, tumour stage, tumour grade, clustering, and age were significantly associated with overall survival (P < 0.01) (Fig. 6A). However, the correlation between BMI and overall survival was not significant. Multivariate Cox regression analysis revealed that the risk score and cancer development were significantly correlated with the survival rate (Fig. 6B). The results revealed that the overall survival of the low-risk group was higher than that of the high-risk group (Fig. 6A-H). Taken together, these findings indicated that the predicting values of GILncSig in prognosis can be considered independent of other clinicopathological parameters.

### 3.6 Establishment and Verification of a Nomogram for Prognosis Prediction in EC

**Figure 7:**
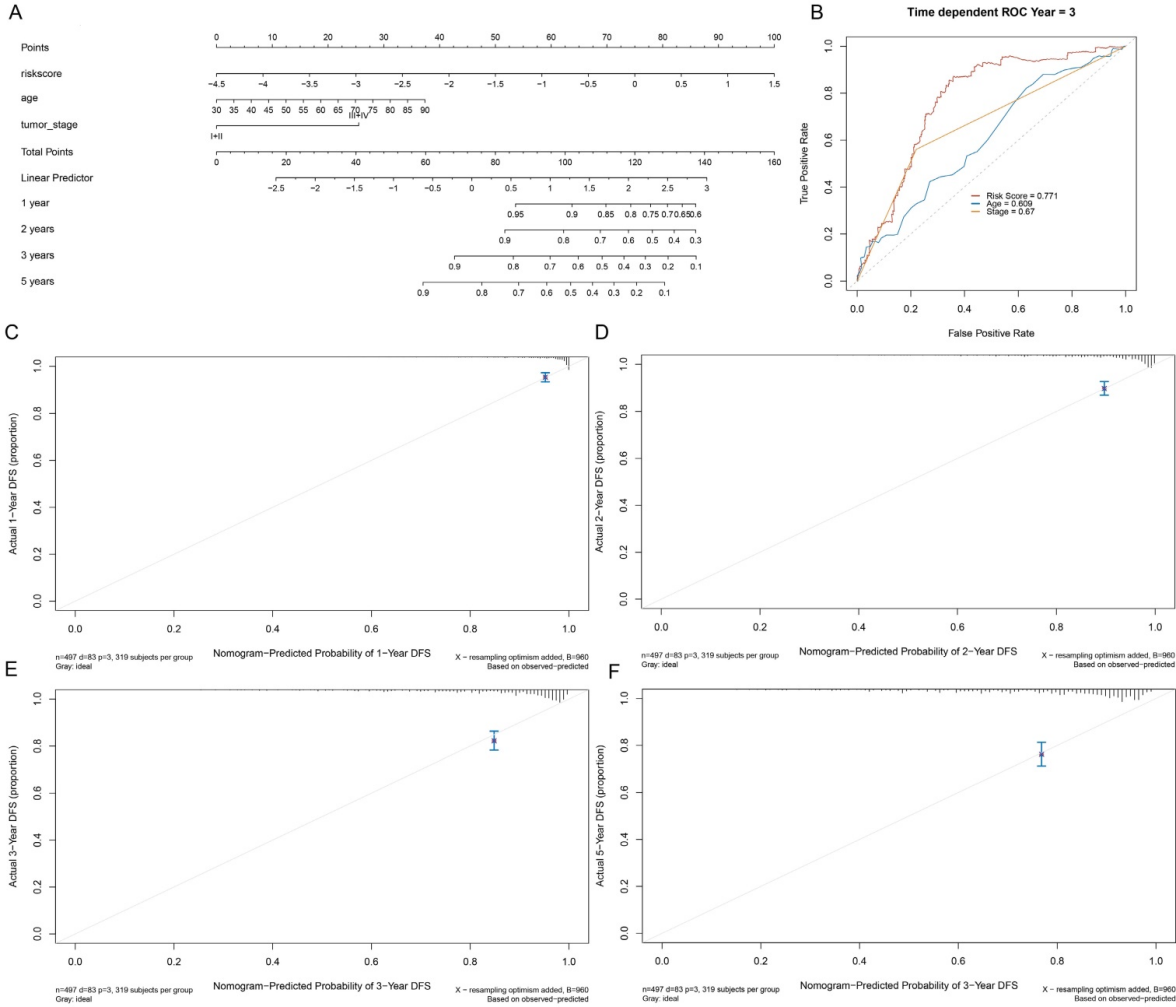
Establishment of a nomogram for prognosis prediction in patients with EC. (A) The nomogram established in the training set for predicting prognosis. (B) ROC curves for 3-year survival prediction of the nomogram. Calibration curve for 1-, 2-, 3-, and 5-year, respectively (C-F).

To validate the prognostic significance of a multi-lncRNA signature, we performed multivariate Cox regression analysis, applying Limma R package to value the accuracy of the risk score and combine GILncSig with prognostic factor, including age, staging, grade and survival rate then construct a statistical nomogram model. The accuracy was verified through the calibration curve. As shown in Fig. 7A and Fig. 7B, the AUC of ROC for 3-year survival predictions was 0.771. The 1-year, 2-year, 3-year, and 5-year survival predictors revealed great consistency between the actual and predicted survival rates of the three data sets (Fig. 7C-F). Overall, these results suggested that the prediction efficacy of the nomogram was enhanced.

**Figure 8:**
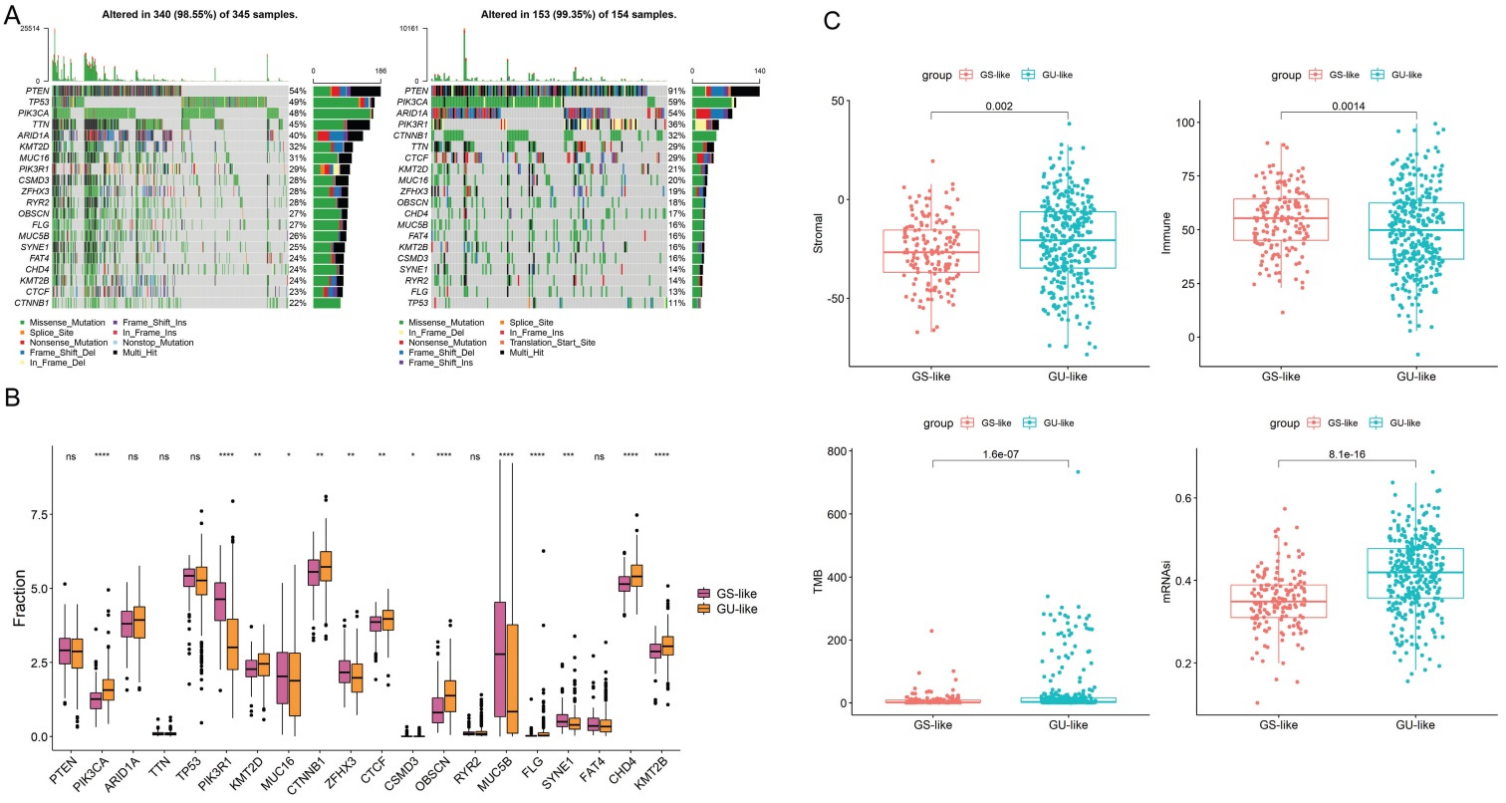
(A) Mutated genes in GU-like group and GS-like group. (B)Mutations in GU-like group(Left) and GS-like group.(C) The stromal score ,immune score, TMB and the stemness index based on mRNA expression (mRNAsi) in GU-like group and GS-like group.

To show the top 20 mutant genes in the GU-like group and GS-like group, cumulative number of somatic mutations per patient was calculated and sorted in the decreasing order. The somatic mutation count of PTEN was the highest in both groups, meanwhile the number of the missense mutations in PIK3CA was the highest in both groups (Fig. 8A-B). High TMB consistently selects for benefit with immune checkpoint blockade (ICB) therapy. Our results show obvious difference in the level of TMB in two group as well as in stromal and immune score (Fig. 8C). Taken together, the GILncSig correlated with genomic mutation rate in EC and can act as an evaluation model of the degree of genome instability.

## 4. Discussion

Genomic instability is a crucial factor that contributes to the acquisition of various human malignancy-related characteristics. Persistent mutations drive tumourigenesis, cancer progression, and resistance to treatment 21. Research has demonstrated that abnormal transcriptional and epigenetic regulation affects the genome stability 22. Studies have investigated mRNA and miRNA markers to determine the extent of genomic instability in cancerous tissues 23. In the past decade, lncRNA expression changes have been shown to promote tumour development and progression and hence can be used as a new tumour biomarker 24, 25. And lncRNAs have been reported to play key roles in EC progression 26. Additionally, lncRNAs and genomic instability exhibit a close relationship. Recent advances in the exploring of functional mechanisms of lncRNAs revealed that lncRNAs are essential for genomic stability, such as NORAD and GUARDIN. Nevertheless, the relationship between genomic instability-related lncRNAs and human EC remains to be fully elucidated. Hence, we propose a GILncSig and examined its prognostic significance in EC. In this study, the EC patients were grouped according to the gene mutation number, and the analysis to screen the differentially expressed genes was performed. Following the multivariate Cox regression analysis, the independent prognostic factors, except for the risk score, were stratified. Among the seven GILncRNAs, PRR34-AS1, FGF14-AS2, GLIS3-AS1, RP11-440D17.3, LINC01224, AF131215.9 were identified as the risk factors for patients prognosis, whereas AC144831.1, HOXVB-AS3, ATP2A1-AS1, MIR210HG, LBX2-AS1, AC092580.4, RP11-760H22.2, RP3-443C4.2 were identified as the protective factors associated with better survival. Among these risk factors, LncRNAPRR34-AS1 has been reported to aggravate the progression of hepatocellular carcinoma 27, GLIS3-AS1 is found to be correlated with the poor prognosis of intraductal papillary mucinous neoplasms 28, and LINC01224 is reported to modulate the malignant transformation in colorectal human cancer, gastric human cancer, ovarian human cancer, and hepatocellular carcinoma 29-32. However, FGF14-AS2 functions as a favourable prognostic biomarker in various human malignancies including breast human malignancy and colorectal human malignancy 33,37,38,39. In this study, MIR210HG was identified as a protective factor, and it has been reported to promote tumour progression in endometrial cancer, non-small cell lung cancer, triple-negative breast cancer, cervical cancer, colorectal cancer, and hepatocellular carcinoma 34-41. Moreover, LBX2-AS1 has been identified as a non-favourable prognostic biomarker in colorectal cancer, ovarian cancer, glioma, and gastric cancer 42, 43. The other lncRNAs, namely RP11-440D17.3, AF131215.9, and AC144831.1, HOXVB-AS3, ATP2A1-AS1, AC092580.4, RP11-760H22.2, and RP3-443C4.2, were studied for the first time in this research. Nevertheless, more studies are warranted to explore their functions in EC prognosis.

In this study, we found 78 gene lncRNAs by screening the expression of lncRNAs among cases with different mutation numbers. These lncRNAs were confirmed to be correlated with genomic instability, which was verified through hierarchical cluster analysis, mutation count, and differential analysis of driving genes responsible for genomic instability. Then the prognostic value of 78 lncRNAs was assessed, and a risk factor score formula composed of 15 lncRNAs was constructed. GILncSig was confirmed as an independent prognostic predictor; patients with a high-risk score were found to often have unfavourable prognosis. Taken together, GILncSig, as a genome instability-derived two lncRNA-based gene signature was proved to stratify patients into high-risk and low-risk groups with significantly different outcome and was validated in multiple independent patient prognostic factors. Additionally, we found a remarkable correlation between the risk score in patients with EC and the tumour mutation pattern, and the high-risk score correlated with high mutation as well as genomic instability. Notably, in different clinical subgroups, risk scores markedly correlated with EC prognosis. These results indicated that the risk factors identified in this study could be the promising markers for prognosis prediction and genomic instability in patients. Finally, a nomogram combining risk factors with tumour staging was constructed in the training set, which further improved the performance and accuracy of the prediction model.

Although we identified GILncSig as a factor for predicting prognosis in EC, our study still has some limitations. Firstly, we only used the data in the TCGA EC database.Therefore, more independent data sets are needed for further verification. Secondly, RP11-440D17.3, AF131215.9, AC144831.1, HOXVB-AS3, ATP2A1-AS1, AC092580.4, RP11-760H22.2, and RP3-443C4.2 associated with genomic instability, which is related to the prognosis of EC have been reported for the first time. Therefore, further studies are required to clarify their roles in EC. Thirdly, more biological experiments are warranted to verify and investigate the mechanism of GILncSig in the genome stability. Currently, our results are being validated in clinical trials and our conclusion would be verified in follow-up studies.

## Figures and Tables

**Table 1:** Clinicopathological information of the patients with EC in the TCGA cohort.

	Type	Number
Os	0	416
	1	83
Age	≥60	330
	<60	167
	NA	2
Stage	I	309
	II	50
	III	115
	IV	25
Grade	G1	93
	G2	109
	G3	288
	High Grade	9
BMI	≥28	319
	<28	151
	NA	29
Pregnancies	0	64
	1	46
	2	112
	3	61
	4+	66
	NA	150
